# Mid‐life occupational hazardous exposures is associated with late‐life dementia risk in the Wisconsin Longitudinal Study

**DOI:** 10.1002/alz.093254

**Published:** 2025-01-09

**Authors:** Victoria J. Williams, Kamil Sicinski, Ralph Trane, Pamela Herd, Sanjay Asthana

**Affiliations:** ^1^ University of Wisconsin‐Madison, Madison, WI USA; ^2^ University of Wisconsin Madison, Madison, WI USA; ^3^ Georgetown Univeristy, Washington DC, DC USA; ^4^ University of Wisconsin‐Madison School of Medicine and Public Health, Madison, WI USA; ^5^ VA Geriatric Research, Education and Clinical Center (GRECC), William S. Middleton Memorial Veterans Hospital, Madison, WI USA

## Abstract

**Background:**

Exposures to environmental toxicants and pollutants occur at various points along the life course, with mounting evidence that late‐life pollution exposure increases risk for neurological disease, including dementia. Although occupational hazards constitute a primary source of modifiable environmental exposure during the working years, there is little research examining the protracted effects of mid‐life occupational exposure on late‐life dementia risk.

**Method:**

This study leveraged life course data from the Wisconsin Longitudinal Study (WLS) to evaluate the effects of mid‐life occupational hazardous exposures on late‐life dementia outcomes. The WLS is a randomly selected population‐based cohort of 1/3 of all 1957 Wisconsin high school graduates and a selected sibling. Occupational hazardous exposures (to solvents and chemicals, pesticides, or airborne particulate matter) was prospectively collected in mid‐life (age ∼54). Dementia status was determined by a clinician consensus‐based approach using comprehensive medical and cognitive date collected in late‐life (age ∼81). Binary logistic regression was used to determine whether midlife occupational exposure predicted late‐life dementia risk, covarying for the effects of age, sex, education, adolescent IQ, ApoE‐4 status, systemic disease burden (hypertension, diabetes, stroke, heart disease), comprehensive smoking history, and occupational education and earnings scores.

**Result:**

3811 WLS participants were included in the analysis, with 523 (13.7%) reporting history of midlife occupational hazardous exposure, and 322 (8.4%) receiving a research diagnosis of dementia. The full binary logistic regression model was statistically significant (Chi^2^(13) = 177.55, p<0.001), accounting for 10.4% of the overall variance in dementia outcome. Midlife occupational exposure significantly predicted dementia risk, such that history of occupational exposure conferred 47.8% increased odds of dementia in late‐life (OR = 1.478 [95%CI: 1.082‐2.020]).

**Conclusion:**

Reported history of mid‐life hazardous occupational exposures significantly increased the odds of all‐cause dementia in the WLS cohort, even after accounting for key confounders including job characteristics that may independently influence dementia risk. Because dementia pathologies often begin accumulating in the brain 20‐30 years prior to the onset of the clinical syndrome, midlife offers a key window for addressing modifiable risk factors to prevent disease. Public policy regulating occupational hazardous exposures in the workplace constitutes a promising strategy to reduce dementia in the general population.

